# Supplementary material for: Impact of Unexpected In-House Major COVID-19 Outbreaks on Depressive Symptoms among Healthcare Workers: A Retrospective Multi-Institutional Study
Source: Int J Environ Res Public Health. 2023 Mar 7;20(6):4718. doi: 10.3390/ijerph20064718 (PMC10049033; doi:10.3390/ijerph20064718)
Supplement: Supplementary file 1 [file ijerph-20-04718-s001.zip › ijerph-2228535-supplementary.pdf]

Table S1. Multiple logistic regression analysis for the PHQ-9 total scores ≥10 compared with <10) by the period of in-house COVID-19 outbreak occurrence

	December 2020 to May 2021 (*n* = 243)				August 2021 to April 2022 (*n* = 283)			
	OR	95% CI		*p*	*OR*	95% CI		*p*
		LL	*UL*			*LL*	*UL*	
Facility type								
Hospital	Ref				Ref			
Nursing home	0.99	0.20	4.81	0.989	1.04	0.53	2.05	0.906
Number of days between outbreak occurrence and response to the screening test	0.99	0.98	1.01	0.497	0.98	0.90	1.07	0.688
Sex								
Woman	Ref				Ref			
Man	1.08	0.51	2.32	0.835	1.10	0.55	2.20	0.781
Age (years)								
20–29	Ref				Ref			
30–39	0.79	0.33	1.88	0.595	0.56	0.26	1.20	0.137
40–49	1.38	0.58	3.28	0.467	1.44	0.67	3.12	0.352
50–59	0.78	0.29	2.12	0.625	1.26	0.57	2.78	0.562
≥60	1.09	0.33	3.67	0.886	0.99	0.29	3.36	0.985
Job type								
Other HCW	Ref				Ref			
Nurse	**2.40**	**1.24**	**4.66**	**0.010**	**2.45**	**1.29**	**4.66**	**0.006**
Nursing home staff	2.01	0.41	9.81	0.388	1.51	0.66	3.46	0.332
COVID-19 infection status								
PCR-negative	Ref				Ref			
PCR-positive	1.81	0.82	4.03	0.145	1.43	0.73	2.80	0.295
Unknown PCR status	1.47	0.35	6.12	0.599	1.54	0.60	3.99	0.370
R^2	0.08				0.11 *			

Note. HCW, healthcare worker; PCR, Polymerase Chain Reaction; PHQ-9, Patient Health Questionnaire-9.
* $p < .05$

Table S2. Multiple logistic regression analysis for the PHQ-9 total scores (≥10 compared with <10) by facility type

	Hospital (n = 405)				Nursing home (n = 121)			
	OR	95% CI		*p*	*OR*	95% CI		*p*
		LL	*UL*			*LL*	*UL*	
Number of days between outbreak occurrence and response to the screening test	0.99	0.98	1.00	0.104	1.02	0.96	1.07	0.594
Sex								
Woman	Ref				Ref			
Man	0.89	0.50	1.59	0.692	2.14	0.79	5.85	0.136
Age (years)								
20–29	Ref				Ref			
30–39	0.62	0.33	1.18	0.144	0.63	0.14	2.72	0.531
40–49	1.36	0.74	2.50	0.329	1.21	0.26	5.70	0.808
50–59	0.92	0.46	1.84	0.820	1.16	0.26	5.24	0.844
≥60	1.13	0.41	3.16	0.810	0.85	0.14	5.27	0.865
Job type								
Other HCW	Ref				Ref			
Nurse	**2.36**	**1.45**	**3.85**	**0.001**	2.95	0.91	9.58	0.073
Nursing home staff	1.53	0.48	4.88	0.469	1.82	0.71	4.64	0.211
COVID-19 infection status								
PCR-negative	Ref				Ref			
PCR-positive	**1.85**	**1.00**	**3.41**	**0.050**	0.84	0.30	2.33	0.741
Unknown PCR status	2.20	0.75	6.49	0.153	0.67	0.22	2.04	0.480
R^2	0.12 **				0.09			

Note. HCW, healthcare worker; PCR, Polymerase Chain Reaction; PHQ-9, Patient Health Questionnaire-9.
** $p < .01$

Table S3. Multiple logistic regression analysis for the PHQ-9 total scores (≥10 compared with <10) by sex

	Woman (*n* = 394)				Man (*n* = 132)			
	OR	95% CI		*p*	*OR*	95% CI		*p*
		LL	*UL*			*LL*	*UL*	
Facility type								
Hospital	Ref				Ref			
Nursing home	0.85	0.44	1.62	0.615	2.53	0.60	10.68	0.208
Number of days between outbreak occurrence and response to the screening test	**0.99**	**0.97**	**1.00**	**0.043**	1.01	0.98	1.03	0.570
Age (years)								
20–29	Ref				Ref			
30–39	0.63	0.31	1.28	0.199	0.53	0.18	1.50	0.230
40–49	1.41	0.75	2.65	0.288	0.89	0.20	3.90	0.875
50–59	1.20	0.62	2.33	0.594	0.33	0.06	1.74	0.192
≥60	0.99	0.40	2.45	0.984	0.87	0.08	9.52	0.912
Job type								
Other HCW	Ref				Ref			
Nurse	**2.71**	**1.62**	**4.52**	**<0.001**	2.67	0.91	7.89	0.075
Nursing home staff	1.78	0.81	3.91	0.149	1.22	0.28	5.33	0.787
COVID-19 infection status								
PCR-negative	Ref				Ref			
PCR-positive	1.27	0.71	2.25	0.418	2.68	0.87	8.30	0.086
Unknown PCR status	1.50	0.64	3.49	0.346	1.35	0.33	5.56	0.679
R^2	0.11 **				0.15			

Note. HCW, healthcare worker; PCR, Polymerase Chain Reaction; PHQ-9, Patient Health Questionnaire-9.
** $p < .01$

Table S4. Multiple logistic regression analysis for the PHQ-9 total scores (≥10 compared with <10) by age

	Aged 20–29 (*n* = 132)				Aged 30–39 (*n* = 143)				Aged 40–49 (*n* = 114)				Aged 50–59 (*n* = 103)				Aged ≥60 (*n* = 34)			
	OR	95% CI		*p*	*OR*	95% CI		*p*	*OR*	95% CI		*OR*	*OR*	95% CI		*p*	*OR*	95% CI		*p*
		LL	*UL*			*LL*	*UL*			*LL*	*UL*			*LL*	*UL*			*LL*	*UL*	
Facility type																				
Hospital	Ref				Ref				Ref				Ref				Ref			
Nursing home	0.48	0.03	6.85	0.587	1.07	0.28	4.05	0.922	0.70	0.22	2.19	0.538	1.27	0.46	3.53	0.643	15.09	0.25	894.35	0.193
Number of days between outbreak occurrence and response to the screening test	1.00	0.97	1.02	0.667	1.00	0.98	1.02	0.971	0.98	0.96	1.01	0.126	0.98	0.94	1.01	0.237	1.07	0.97	1.18	0.155
Sex																				
Woman	Ref				Ref				Ref				Ref				Ref			
Man	1.11	0.47	2.61	0.818	1.18	0.49	2.83	0.716	1.16	0.33	4.14	0.819	0.47	0.11	2.07	0.318	8.03	0.83	77.60	0.072
Job type																				
Other HCW	Ref				Ref				Ref				Ref				Ref			
Nurse	1.92	0.80	4.63	0.145	2.05	0.76	5.51	0.155	2.33	0.88	6.15	0.088	1.84	0.63	5.41	0.267	**917.50**	**14.67**	**57383.72**	**0.001**
Nursing home staff	5.54	0.34	89.75	0.228	1.87	0.44	7.89	0.397	2.37	0.54	10.35	0.253	0.78	0.19	3.14	0.726	18.12	1.17	279.89	0.038
COVID-19 infection status																				
PCR-negative	Ref				Ref				Ref				Ref				Ref			
PCR-positive	1.17	0.33	4.17	0.809	1.91	0.69	5.30	0.211	1.08	0.37	3.13	0.886	3.03	0.98	9.35	0.053	0.07	0.00	6.22	0.245
Unknown PCR status	1.25	0.16	9.58	0.831	1.36	0.24	7.58	0.726	1.49	0.44	5.01	0.518	2.06	0.45	9.49	0.354	0.37	0.00	27.59	0.651
R^2	0.05				0.06				0.10				0.17				0.61	*		

Note. HCW, healthcare worker; PCR, Polymerase Chain Reaction; PHQ-9, Patient Health Questionnaire-9.

* $p < .05$

Table S5. Multiple logistic regression analysis for the PHQ-9 total scores (≥10 compared with <10) by job type

	Nurse (*n* =214)				Nursing home staff (*n* =71)				Other HCW (*n* = 241)			
	OR	95% CI		*p*	*OR*	95% CI		*p*	*OR*	95% CI		*p*
		LL	*UL*			*LL*	*UL*			*LL*	*UL*	
Facility type												
Hospital	Ref				Ref				Ref			
Nursing home	1.00	0.39	2.52	0.992	1.19	0.32	4.38	0.794	1.35	0.50	3.66	0.554
Number of days between outbreak occurrence and response to the screening test	0.99	0.97	1.01	0.226	1.01	0.92	1.11	0.801	1.00	0.98	1.01	0.807
Sex												
Woman	Ref				Ref				Ref			
Man	0.95	0.36	2.53	0.919	1.25	0.34	4.60	0.742	1.04	0.54	2.00	0.906
Age (years)												
20–29	Ref				Ref				Ref			
30–39	0.63	0.26	1.53	0.311	0.38	0.07	2.17	0.278	0.64	0.27	1.51	0.304
40–49	1.43	0.63	3.25	0.390	0.80	0.12	5.48	0.824	1.21	0.46	3.15	0.699
50–59	1.21	0.47	3.11	0.693	0.45	0.08	2.69	0.383	0.95	0.36	2.54	0.921
≥60	2.37	0.64	8.71	0.194	0.30	0.03	2.95	0.303	0.29	0.03	2.90	0.294
COVID-19 infection status												
PCR-negative	Ref				Ref				Ref			
PCR-positive	1.84	0.89	3.78	0.098	0.76	0.21	2.76	0.676	1.45	0.55	3.79	0.453
Unknown PCR status	2.96	0.76	11.48	0.116	0.70	0.18	2.71	0.600	1.00	0.24	4.24	0.999
R^2	0.08				0.05				0.04			

Note. HCW, healthcare worker; PCR, Polymerase Chain Reaction; PHQ-9, Patient Health Questionnaire-9.

Table S6. Multiple logistic regression analysis for the PHQ-9 total scores (≥10 compared with <10) by COVID-19 infection status

	PCR-negative (*n* = 377)				PCR-positive (*n* = 102)				Unknown (*n* = 47)			
	OR	95% CI		*p*	*OR*	95% CI		*p*	*OR*	95% CI		*p*
		LL	*UL*			*LL*	*UL*			*LL*	*UL*	
Facility type												
Hospital	Ref				Ref				Ref			
Nursing home	1.35	0.62	2.92	0.452	1.17	0.36	3.81	0.800	0.64	0.08	5.51	0.688
Number of days between outbreak occurrence and response to the screening test	0.99	0.97	1.00	0.076	1.00	0.98	1.03	0.933	0.97	0.91	1.03	0.285
Sex												
Woman	Ref				Ref				Ref			
Man	0.81	0.43	1.51	0.507	3.17	0.82	12.24	0.095	0.88	0.19	4.03	0.865
Age (years)												
20–29	Ref				Ref				Ref			
30–39	0.53	0.27	1.06	0.074	0.69	0.18	2.68	0.589	1.13	0.06	20.82	0.935
40–49	1.29	0.68	2.47	0.436	1.13	0.29	4.42	0.858	2.90	0.13	65.11	0.502
50–59	0.72	0.34	1.51	0.384	1.78	0.49	6.43	0.378	2.44	0.09	67.38	0.599
≥60	1.09	0.39	3.06	0.864	0.72	0.10	5.29	0.749	1.75	0.05	60.18	0.758
Job type												
Other HCW	Ref				Ref				Ref			
Nurse	**2.16**	**1.29**	**3.60**	**0.003**	**4.80**	**1.18**	**19.58**	**0.029**	5.90	0.56	62.41	0.140
Nursing home staff	1.92	0.73	5.02	0.184	1.84	0.42	8.11	0.422	1.74	0.25	12.06	0.574
R^2	0.11	**			0.13				0.21			

Note. HCW, healthcare worker; PCR, Polymerase Chain Reaction; PHQ-9, Patient Health Questionnaire-9.
** $p < .01$

Table S7. Multiple logistic regression analysis for the item nine scores of PHQ-9 (≥1 compared with <1) by the period of in-house COVID-19 outbreak occurrence

	December 2020 to May 2021 (*n* = 243)				August 2021 to April 2022 (*n* = 283)			
	OR	95% CI		*p*	*OR*	95% CI		*p*
		LL	*UL*			*LL*	*UL*	
Facility type								
Hospital	Ref				Ref			
Nursing home	4.36	0.50	38.25	0.183	1.30	0.50	3.39	0.591
Number of days between outbreak occurrence and response to the screening test	1.01	0.97	1.04	0.767	1.00	0.88	1.15	0.982
Sex								
Woman	Ref				Ref			
Man	1.44	0.53	3.92	0.478	1.06	0.41	2.76	0.907
Age (years)								
20–29	Ref				Ref			
30–39	0.82	0.21	3.20	0.777	**0.32**	**0.12**	**0.89**	**0.029**
40–49	0.76	0.16	3.63	0.726	0.66	0.25	1.71	0.392
50–59	N/A				**0.28**	**0.09**	**0.89**	**0.031**
≥60	1.46	0.19	11.19	0.715	0.21	0.02	2.07	0.183
Job type								
Other HCW	Ref				Ref			
Nurse	**1.75**	**0.63**	**4.87**	**0.280**	**3.08**	**1.18**	**8.05**	**0.021**
Nursing home staff	N/A				**3.95**	**1.18**	**13.26**	**0.026**
COVID-19 infection status								
PCR-negative	Ref				Ref			
PCR-positive	1.63	0.40	6.68	0.497	1.43	0.65	3.17	0.372
Unknown PCR status	0.50	0.03	7.73	0.620	0.18	0.02	1.63	0.127
R^2	0.96				0.19 *			

Note. HCW, healthcare worker; PCR, Polymerase Chain Reaction; PHQ-9, Patient Health Questionnaire-9.
* *p* < .05

Table S8. Multiple logistic regression analysis for the item nine scores of PHQ-9 (≥1 compared with <1) by facility type

	Hospital (n = 405)				Nursing home (n = 121)			
	OR	95% CI		*p*	*OR*	95% CI		*p*
		LL	*UL*			*LL*	*UL*	
Number of days between outbreak occurrence and response to the screening test	0.98	0.96	1.00	0.111	0.98	0.86	1.11	0.729
Sex								
Woman	Ref				Ref			
Man	1.02	0.44	2.38	0.963	3.22	0.68	15.21	0.140
Age (years)								
20–29	Ref				Ref			
30–39	0.39	0.15	1.02	0.054	0.38	0.06	2.51	0.316
40–49	0.66	0.28	1.56	0.342	0.91	0.12	6.90	0.927
50–59	**0.14**	**0.03**	**0.58**	**0.007**	0.36	0.04	3.00	0.346
≥60	0.36	0.05	2.70	0.324	0.49	0.04	5.56	0.565
Job type								
Other HCW	Ref				Ref			
Nurse	**2.50**	**1.14**	**5.48**	**0.022**	4.00	0.78	20.59	0.097
Nursing home staff	**11.10**	**2.54**	**48.51**	**0.001**	1.61	0.41	6.42	0.497
COVID-19 infection status								
PCR-negative	Ref				Ref			
PCR-positive	1.71	0.72	4.02	0.223	1.34	0.37	4.89	0.659
Unknown PCR status	0.31	0.03	3.73	0.354	0.21	0.02	2.42	0.208
R^2	0.20	**			0.29			

Note. HCW, healthcare worker; PCR, Polymerase Chain Reaction; PHQ-9, Patient Health Questionnaire-9.

** $p < .01$

Table S9. Multiple logistic regression analysis for the item nine scores of PHQ-9 (≥1 compared with <1) by sex

	Woman (n = 394)				Man (n = 132)			
	OR	95% CI		*p*	*OR*	95% CI		*p*
		LL	*UL*			*LL*	*UL*	
Facility type								
Hospital	Ref				Ref			
Nursing home	1.46	0.44	4.85	0.539	1.62	0.22	11.69	0.634
Number of days between outbreak occurrence and response to the screening test	**0.97**	**0.95**	**0.99**	**0.017**	1.00	0.97	1.04	0.861
Age (years)								
20–29	Ref				Ref			
30–39	**0.25**	**0.09**	**0.70**	**0.009**	0.91	0.23	3.68	0.900
40–49	0.57	0.25	1.33	0.194	1.52	0.29	7.98	0.620
50–59	**0.19**	**0.07**	**0.56**	**0.002**	0.92	0.10	8.59	0.940
≥60	0.27	0.05	1.37	0.114	1.40	0.03	69.22	0.867
Job type								
Other HCW	Ref				Ref			
Nurse	**2.73**	**1.21**	**6.17**	**0.016**	2.78	0.73	10.59	0.135
Nursing home staff	2.32	0.52	10.42	0.272	5.45	0.66	44.70	0.114
COVID-19 infection status								
PCR-negative	Ref				Ref			
PCR-positive	1.19	0.52	2.74	0.676	2.31	0.56	9.51	0.247
Unknown PCR status	0.23	0.03	1.96	0.177	0.34	0.02	6.88	0.484
R^2	0.23 **				0.17			

Note. HCW, healthcare worker; PCR, Polymerase Chain Reaction; PHQ-9, Patient Health Questionnaire-9.
** $p < .01$

Table S10. Multiple logistic regression analysis for the item nine scores of PHQ-9 (≥1 compared with <1) by age

	Aged 20–29 (*n* = 132)				Aged 30–39 (*n* = 143)				Aged 40–49 (*n* = 114)				Aged 50–59 (*n* = 103)			
	OR	95% CI		*p*	*OR*	95% CI		*p*	*OR*	95% CI		*OR*	*OR*	95% CI		*p*
		LL	*UL*			*LL*	*UL*			*LL*	*UL*			*LL*	*UL*	
Facility type																
Hospital	Ref				Ref				Ref				Ref			
Nursing home	2.53	0.20	32.06	0.473	0.80	0.20	3.18	0.756	0.83	0.11	6.04	0.856	1.70	0.23	12.61	0.601
Number of days between outbreak occurrence and response to the screening test	**0.96**	**0.93**	**0.99**	**0.022**	1.01	0.97	1.06	0.501	0.97	0.93	1.01	0.127	0.85	0.72	1.01	0.068
Sex																
Woman	Ref				Ref				Ref				Ref			
Man	0.57	0.18	1.83	0.348	2.03	0.59	7.03	0.265	1.90	0.29	12.55	0.506	2.99	0.41	21.89	0.280
Job type																
Other HCW	Ref				Ref				Ref				Ref			
Nurse	**4.67**	**1.31**	**16.61**	**0.017**	1.61	0.29	9.10	0.587	2.53	0.37	17.40	0.344	1.29	0.21	7.99	0.785
Nursing home staff	1.26	0.08	20.27	0.870	**10.50**	**2.42**	**45.69**	**0.002**	5.07	0.47	54.47	0.180	1.79	0.26	12.23	0.553
COVID-19 infection status																
PCR-negative	Ref				Ref				Ref				Ref			
PCR-positive	1.72	0.50	6.00	0.392	0.91	0.17	4.86	0.912	**3.57**	**1.07**	**11.95**	**0.039**	1.48	0.24	9.00	0.669
Unknown PCR status	0.54	0.04	6.79	0.632	0.49	0.02	10.05	0.644	N/A				N/A			
R^2	0.24				0.16				0.27				0.66			

Note. HCW, healthcare worker; PCR, Polymerase Chain Reaction; PHQ-9, Patient Health Questionnaire-9. Analysis could not be done for aged ≥60 due to small number of cases.

Table S11. Multiple logistic regression analysis for the item nine scores of PHQ-9 (≥1 compared with <1) by job type

	Nurse (*n* =214)				Nursing home staff (*n* =71)				Other HCW (*n* = 241)			
	OR	95% CI		*p*	*OR*	95% CI		*p*	*OR*	95% CI		*p*
		LL	*UL*			*LL*	*UL*			*LL*	*UL*	
Facility type												
Hospital	Ref				Ref				Ref			
Nursing home	3.34	0.91	12.24	0.069	**0.17**	**0.04**	**0.76**	**0.021**	2.16	0.68	6.90	0.192
Number of days between outbreak occurrence and response to the screening test	**0.97**	**0.95**	**1.00**	**0.028**	0.92	0.83	1.01	0.093	1.00	0.97	1.03	0.968
Sex												
Woman	Ref				Ref				Ref			
Man	0.80	0.23	2.82	0.731	5.26	0.71	38.95	0.104	1.08	0.43	2.73	0.865
Age (years)												
20–29	Ref				Ref				Ref			
30–39	**0.19**	**0.04**	**0.81**	**0.026**	1.72	0.23	12.80	0.594	0.51	0.12	2.10	0.350
40–49	0.40	0.13	1.19	0.099	**6.10**	**1.10**	**33.80**	**0.038**	0.74	0.14	3.90	0.722
50–59	**0.09**	**0.02**	**0.47**	**0.004**	0.80	0.14	4.66	0.804	0.56	0.10	3.04	0.498
≥60	0.35	0.07	1.86	0.219	N/A				0.95	0.07	13.44	0.970
COVID-19 infection status												
PCR-negative	Ref				Ref				Ref			
PCR-positive	1.90	0.70	5.17	0.207	3.84	0.88	16.66	0.073	1.45	0.39	5.38	0.579
Unknown PCR status	0.42	0.04	4.28	0.462	N/A				0.51	0.05	5.23	0.567
R^2	0.19				0.35				0.05			

Note. HCW, healthcare worker; PCR, Polymerase Chain Reaction; PHQ-9, Patient Health Questionnaire-9.

Table S12. Multiple logistic regression analysis for the item nine scores of PHQ-9 (≥1 compared with <1) by COVID-19 infection status

	PCR-negative (*n* = 377)				PCR-positive (*n* = 102)				Unknown PCR status (*n* = 47)			
	OR	95% CI		*p*	*OR*	95% CI		*p*	*OR*	95% CI		*p*
		LL	*UL*			*LL*	*UL*			*LL*	*UL*	
Facility type												
Hospital	Ref				Ref				Ref			
Nursing home	1.51	0.45	5.10	0.505	1.09	0.11	11.05	0.940	0.79	0.10	6.13	0.820
Number of days between outbreak occurrence and response to the screening test	0.99	0.96	1.01	0.203	0.97	0.93	1.02	0.218	0.99	0.93	1.05	0.770
Sex												
Woman	Ref				Ref				Ref			
Man	0.84	0.32	2.20	0.719	**6.95**	**1.50**	**32.18**	**0.013**	1.34	0.33	5.51	0.683
Age (years)												
20–29	Ref				Ref				Ref			
30–39	0.42	0.16	1.10	0.078	0.18	0.02	1.43	0.106	3.51	0.16	77.58	0.426
40–49	0.60	0.23	1.56	0.298	1.42	0.30	6.68	0.656	N/A			
50–59	**0.30**	**0.09**	**0.98**	**0.046**	0.23	0.03	1.65	0.145	N/A			
≥60	0.45	0.07	2.95	0.405	0.46	0.02	8.66	0.602	N/A			
Job type												
Other HCW	Ref				Ref				Ref			
Nurse	**2.43**	**1.10**	**5.38**	**0.028**	5.36	0.98	29.28	0.052	3.31	0.40	27.54	0.269
Nursing home staff	3.71	0.84	16.44	0.084	4.49	0.57	35.01	0.152	N/A			
R^2	0.15				0.29				0.17			

Note. HCW, healthcare worker; PCR, Polymerase Chain Reaction; PHQ-9, Patient Health Questionnaire-9.
